# Estimation of medium effects on equilibrium constants in moderate and high ionic strength solutions at elevated temperatures by using specific interaction theory (SIT): Interaction coefficients involving Cl, OH^- ^and Ac^- ^up to 200°C and 400 bars

**DOI:** 10.1186/1467-4866-7-4

**Published:** 2006-05-23

**Authors:** Yongliang Xiong

**Affiliations:** 1 Sandia National Laboratories, Carlsbad Programs Group, 4100 National Parks Highway, Carlsbad, NM 88220, USA

## Abstract

In this study, a series of interaction coefficients of the Brønsted-Guggenheim-Scatchard specific interaction theory (SIT) have been estimated up to 200°C and 400 bars. The interaction coefficients involving Cl^- ^estimated include ε(H^+^, Cl^-^), ε(Na^+^, Cl^-^), ε(Ag^+^, Cl^-^), ε(Na^+^, AgCl_2 _^-^), ε(Mg^2+^, Cl^-^), ε(Ca^2+^, Cl^-^), ε(Sr^2+^, Cl^-^), ε(Ba^2+^, Cl^-^), ε(Sm^3+^, Cl^-^), ε(Eu^3+^, Cl^-^), ε(Gd^3+^, Cl^-^), and ε(GdAc^2+^, Cl^-^). The interaction coefficients involving OH^- ^estimated include ε(Li^+^, OH^-^), ε(K^+^, OH^-^), ε(Na^+^, OH^-^), ε(Cs^+^, OH^-^), ε(Sr^2+^, OH^-^), and ε(Ba^2+^, OH^-^). In addition, the interaction coefficients of ε(Na^+^, Ac^-^) and ε(Ca^2+^, Ac^-^) have also been estimated. The bulk of interaction coefficients presented in this study has been evaluated from the mean activity coefficients. A few of them have been estimated from the potentiometric and solubility studies.

The above interaction coefficients are tested against both experimental mean activity coefficients and equilibrium quotients. Predicted mean activity coefficients are in satisfactory agreement with experimental data. Predicted equilibrium quotients are in very good agreement with experimental values.

Based upon its relatively rapid attainment of equilibrium and the ease of determining magnesium concentrations, this study also proposes that the solubility of brucite can be used as a pH (pcH) buffer/sensor for experimental systems in NaCl solutions up to 200°C by employing the predicted solubility quotients of brucite in conjunction with the dissociation quotients of water and the first hydrolysis quotients of Mg^2+^, all in NaCl solutions.

## Introduction

Knowledge of medium effects on thermodynamics in concentrated solutions is fundamentally important to the thermodynamic modeling in many fields ranging from experimental systems in aqueous solutions to hydrothermal ore deposits of the natural systems. In two recent, detailed reviews [1,2], several models which can handle moderate to high ionic strength solutions are surveyed. Those models surveyed include the Pitzer equations [3], the Brønsted-Guggenheim-Scatchard specific interaction theory (SIT) [4-6], the Bromley model [7], and the Helgeson activity coefficient model [8]. In addition, although not surveyed in the above two reviews, the commonly used B dot equation [9] in geochemistry is valid to the ionic strength of 1.0 m at most [10].

Because they have a large number of adjustable parameters, the Pitzer equations are excellent in fitting the experimental data in highly concentrated solutions as well as in diluted solutions [11]. Therefore, the Pitzer equations can accurately reproduce activity coefficients and other thermodynamic properties at high ionic strength up to the saturation of most salts.

The SIT model is most useful in the ionic strength range up to 3.5–4.0 m [e.g, [12-15]], and successful applications of the SIT model at 25°C in NaCl solutions up to the saturation of halite have also been demonstrated [e.g., [16]]. The SIT model can be regarded as a simplified version of the Pitzer formalism without consideration of triple interactions and interactions between ions of the same charge sign. Therefore, the Pitzer formalism is certainly superior to the SIT model. The shortcoming of the SIT model is its rather low accuracy in reproduction of mean activity coefficients in comparison with Pitzer model [2]. However, the error is usually less than 10% at ionic strength up to 6–10 m at 25°C [2].

The Bromley model is similar to the SIT model, but it takes the concentration dependence of second virial coefficients into consideration. Accordingly, the Bromley model fits experimental data slightly better than the SIT model does [2]. However, Wang et al. [2] also pointed out that even though the Bromley model has a more complicated analytical form than the SIT model, both the Bromley and SIT models reproduce experimental data with practically equal quality according to their extensive evaluation.

As stated by Grenthe et al. [1] and Wang et al. [2], the Helgeson activity coefficient model is actually a one-parameter equation, and it has the same accuracy as that of the SIT model. Nevertheless, the validity of the assumptions of the Helgeson activity coefficient model is not clear. Furthermore, the usage of different values of the ion size parameters (a_j_) for different ions and electrolytes is considered as an obvious drawback of the model, because it creates difficulties in employing the model to mixtures of electrolytes, and results in the violation of cross-differential relations [1,2].

In investigations of systems where complex formation takes place, a method of constant ionic medium is usually adopted. As pointed out by Wang et al. [2], there are difficulties in determination of activity coefficients of reaction species in a constant ionic medium. Usually only a value of equilibrium constant in a certain medium can be determined, and the number of equilibrium constants obtained is generally small. Second, the accuracy of equilibrium constants is relatively low in comparison with that of mean activity coefficients and osmotic coefficients. Accordingly, owing to these two facts, it is sensible to use an activity model with fewer parameters when dealing with experimental equilibrium constants, as it is often impractical to determine more than one or two empirical parameters from a small number of such constants with limited accuracy. The Pitzer equations are widely utilized to treat a number of data with an accuracy better than 0.5 relative percent. However, when describing equilibrium constants as a function of ionic strength, the Pitzer formalism commonly has an accuracy up to a relative percentage of 10–50, which results in such an ill-conditioned assignment that a unique set of the Pitzer parameters cannot be determined [2].

Grenthe et al. [1] provided detailed examples for comparison of the SIT model with the Pitzer formalism in the description of medium effects on equilibrium constants at 25°C. Their comparison indicates that the less-parameterized SIT model gives quite reasonable estimations of equilibrium constants in different media at various ionic strengths, provided that the necessary interaction coefficients are known. They concluded that the simple one-parameter SIT model reproduces the experimental equilibrium data very well.

The above brief descriptions suggest that when the required Pitzer parameters are evaluated accurately from extensive data, the Pitzer model is a preferred, standard method in presentation of experimentally determined thermodynamic properties of electrolytes. However, the SIT model, because of its advantages in mathematical simplicity and its less-parameterized nature, may find applications when the experimental data are less extensive, or the accuracy provided by the SIT model is deemed to be satisfactory, or in systems where complex formation occurs. This is especially true in cases in treatment of equilibrium constants. Consequently, the SIT model has the potential to become a useful method to estimate medium effects on equilibrium constants in concentrated solution in high temperature aqueous geochemistry and chemistry.

However, few studies have addressed SIT interaction coefficients at elevated temperatures. Giffaut et al. [17] estimated the interaction coefficients of ε(H^+^, Cl^-^), and ε(Sr^2+^, Cl^-^) up to 70°C, and ε(Li^+^, Cl^-^), ε(Na^+^, Cl^-^), and ε(K^+^, Cl^-^) up to 150°C. Bretti et al. [18] estimated the interaction coefficients of ε(H^+^, Cl^-^) up to 60°C. It is clear that interaction coefficients of Cl^- ^with geochemically important metal ions such as Mg^2+^, Ca^2+^, and Ba^2+ ^are lacking, and so are interaction coefficients with OH^-^. In addition, those interaction coefficients mentioned in the above studies were not tested against experimental data. Therefore, the main objective of this study is to estimate a series of internally consistent interaction coefficients involving Cl^- ^and OH^- ^up to 200°C. Interaction coefficients involving Cl^- ^are especially of geochemical importance because of its ubiquity in geological fluids.

The intent of this study and future studies of this aspect is to provide experimental aqueous geochemists and high-temperature geochemical modelers a simple means in assessing ionic medium effects on equilibrium constants of various reactions including complex formation reactions at elevated temperatures at ionic strength higher than 1.0 m. Currently, most experimental work on equilibrium constants at elevated temperatures is usually limited up to ionic strength of 1.0 m, which is the limit of the B dot equation. It should be stressed that the Pitzer formalism should be used to treat high precision activity and osmotic coefficients at elevated temperatures, if they are available.

## Estimation of interaction coefficients and their temperature dependence expressions

### Interaction coefficients estimated from mean activity coefficients

In this study, the bulk of interaction coefficients are evaluated from mean activity coefficients of electrolytes. The analytical expression for evaluation of interaction coefficients from mean activity coefficient data of electrolytes, using γ_±, HCl _as an example, is as follows [14]:

2log γ_±, HCl _= -(Z_+_)^2^D + ε(H^+^, Cl^-^) m_HCl _-(Z_-_)^2^D + ε(Cl^-^, H^+^) m_HCl _    (1)

where D is the Debye-Hückel term given by the following expression:



where *A*_γ _is Debye-Hückel slopes for activity coefficient, *Z*_+ _and *Z*_- _are charges of the cation and anion, respectively; ρ the minimum distance of approach between ions [12], and *I *ionic strength on the molal scale. Different values ranging from 1.0 [5] to 1.6 [19] have been proposed for ρ. In this study, ρ is assigned to be 1.5 to follow the convention of Scatchard [6] (p. 145), and numerous studies have adopted this value [e.g., [2,12-14,18,20]]. However, as stated by Grenthe et al. [14], the variation in ñ (Ba_j _term in [14]) values used in the Debye-Hückel term does not represent an uncertainty range, but rather indicates covariation of the parameters ñ and ε(j, k) such that several different combinations of these parameters may reproduce equally well the measured mean activity coefficients of a given electrolyte. The Debye-Hückel slopes for activity coefficient (A_γ_) are temperature and pressure dependent. In this study, the Debye-Hückel slopes for activity coefficient at various temperatures at 1 bar (below 100°C) and saturated vapor pressures (P_SAT_) (100°C and above) are from Helgeson et al. [8], and those at various temperatures at the constant pressure of 200 or 400 bars are from Bradley and Pitzer [21]. The Debye-Hückel slopes from Helgeson et al. [8] at 1 bar and saturated vapor pressures are precise to four decimal places, and those from Bradley and Pitzer [21] at various pressures including saturated vapor pressures are precise to three decimal places. The Debye-Hückel slopes from Helgeson et al. [8] at 0°C–40°C at 1 bar are identical to those of Bradley and Pitzer [21] in the same temperature range at saturated vapor pressures. The Debye-Hückel slopes from these two sources at saturated vapor pressures differ by up to 0.002 in the temperature range from 50°C to 200°C.

Rearranging Eq. (1), we have

log γ_±, HCl _+ D = ε(H^+^, Cl^-^) m_HCl _    (3)

**Figure 1:**
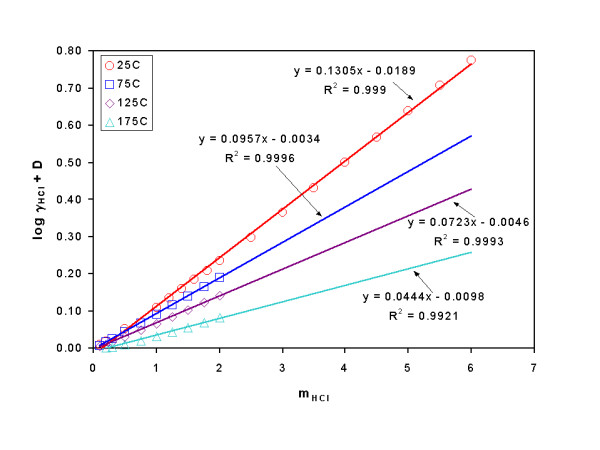
A plot of log γ_±, HCl _+ D versus m_HCl _at 25°C, 75°C, 125°C and 175°C. The slope is the interaction coefficient ε(H^+^, Cl^-^).

According to Eq. (3), by plotting m_HCl _versus log γ_±, HCl _+ D, the slope will be ε(H^+^, Cl^-^), and the linearity of fitting will indicate the ionic strength range for validity of ε(H^+^, Cl^-^). As an example of such evaluation, the plot at 25°C, 75°C, 125°C and 175°C is illustrated in Figure 1.

By following the above methodology, interaction coefficients are systematically evaluated (Table 1). The interaction coefficients of ε(H^+^, Cl^-^) at saturated vapor pressures (P_SAT_) are evaluated from the mean activity coefficients of HCl from the compilation of Robinson and Stokes [22], the electromotive force measurements of Greeley et al. [23] and isopiestic investigations of Holmes et al. [24]. The interaction coefficients of ε(H^+^, Cl^-^) at 400 bars are evaluated from the mean activity coefficients of HCl from the isopiestic data of Holmesetal. [24]. The comparison of ε(H^+^, Cl^-^) at P_SAT _with those at 400 bars reveals that there is a systematic increase in interaction coefficient with pressure, although such increase is within the uncertainty of evaluation.

**Table 1:** Interaction coefficients involving Cl^-^, OH^- ^and Ac^- ^evaluated by this study at various temperatures

Type of Interaction Coefficient	T, K	Value	2σ of ε	Linearity of Fitting (Square of Linear Correlation Coefficient, R^2^)	Ionic Strength, m	Pressure	Experimental Data Sources for Evaluation
ε(H^+^, Cl^-^)	273.15	0.1292	0.0013	0.9998	0.1–2.0	P_SAT_^A^	Holmes et al. [24]
	298.15	0.1305	0.0022	0.9990	0.1–6.0	P_SAT_	Robinson and Stokes [22]; Greeley et al. [23]; Holmes et al. [24]
	348.15	0.0957	0.0013	0.9996	0.1–2.0	P_SAT_	Holmes et al. [24]
	398.15	0.0723	0.0014	0.9993	0.1–2.0	P_SAT_	Holmes et al. [24]
	448.15	0.0444	0.0028	0.9921	0.1–2.0	P_SAT_	Holmes et al. [24]
							
	273.15	0.1304	0.0012	0.9998	0.1–2.0	400 bars	Holmes et al. [24]
	298.15	0.1198	0.0010	0.9998	0.1–2.0	400 bars	
	348.15	0.0986	0.0007	0.9999	0.1–2.0	400 bars	
	398.15	0.0766	0.0007	0.9998	0.1–2.0	400 bars	
	448.15	0.0512	0.0013	0.9987	0.1–2.0	400 bars	

ε(Na^+^, Cl^-^)	273.15	0.0312	0.0028	0.9809	0.5–3.5	P_SAT_	Gibbard et al. [25]
	298.15	0.0471	0.0017	0.9932	0.5–6.0	P_SAT_	
	348.15	0.0467	0.0007	0.9990	0.5–6.0	P_SAT_	
	373.15	0.0432	0.0005	0.9993	0.5–6.0	P_SAT_	
	398.15	0.0389	0.0004	0.9996	0.5–6.0	P_SAT_	
	423.15	0.0338	0.0003	0.9996	0.5–6.0	P_SAT_	
	448.15	0.0279	0.0005	0.9984	0.5–6.0	P_SAT_	
	473.15	0.0210	0.0006	0.9964	0.5–6.0	P_SAT_	

ε(Na^+^, AgCl_2 _^-^)	373.15	-0.02	0.03	0.9821	0.5–3.0	P_SAT_	Seward [41]
	423.15	-0.05	0.03	0.9807	0.5–3.0	P_SAT_	
	470.15	-0.07	0.03	0.9832	0.5–3.0	P_SAT_	

ε(Ag^+^, Cl^-^)	373.15	0.08	0.01	0.9842	0.5–3.0	P_SAT_	Seward [41]
	423.15	0.11	0.02	0.9825	0.5–3.0	P_SAT_	
	470.15	0.13	0.02	0.9840	0.5–3.0	P_SAT_	

ε(Mg^2+^, Cl^-^)	273.15	0.232	0.023	0.9959	0.3–6	P_SAT_	Holmes and Mesmer [27]
	298.15	0.209	0.022	0.9952	0.3–6	P_SAT_	
	323.15	0.191	0.020	0.9951	0.3–6	P_SAT_	
	373.15	0.154	0.019	0.9936	0.3–6	P_SAT_	
	423.15	0.115	0.018	0.9890	0.3–6	P_SAT_	
	473.15	0.075	0.037	0.9738	0.3–6	P_SAT_	
							
	273.15	0.230	0.024	0.9954	0.3–6	200 bars	
	298.15	0.213	0.022	0.9956	0.3–6	200 bars	
	323.15	0.194	0.019	0.9960	0.3–6	200 bars	
	373.15	0.157	0.019	0.9939	0.3–6	200 bars	
	423.15	0.119	0.019	0.9898	0.3–6	200 bars	
	473.15	0.078	0.018	0.9781	0.3–6	200 bars	
							
	273.15	0.235	0.024	0.9957	0.3–6	400 bars	
	298.15	0.217	0.021	0.9959	0.3–6	400 bars	
	323.15	0.198	0.020	0.9958	0.3–6	400 bars	
	373.15	0.162	0.019	0.9944	0.3–6	400 bars	
	423.15	0.126	0.018	0.9909	0.3–6	400 bars	
	473.15	0.087	0.017	0.9824	0.3–6	400 bars	

ε(Ca^2+^, Cl^-^)	273.15	0.166	0.023	0.9922	0.3–6	P_SAT_	Holmes and Mesmer [27]
	298.15	0.161	0.021	0.9936	0.3–6	P_SAT_	Robinson and Stokes [22], Holmes and Mesmer [27]
							
	323.15	0.152	0.017	0.9945	0.3–6	P_SAT_	Holmes and Mesmer [27]
	373.15	0.124	0.013	0.9952	0.3–6	P_SAT_	
	423.15	0.088	0.011	0.9933	0.3–6	P_SAT_	
	473.15	0.046	0.011	0.9754	0.3–6	P_SAT_	
							
	273.15	0.170	0.022	0.9928	0.3–6	200 bars	Holmes and Mesmer [27]
	298.15	0.165	0.019	0.9942	0.3–6	200 bars	
	323.15	0.155	0.017	0.9950	0.3–6	200 bars	
	373.15	0.127	0.013	0.9955	0.3–6	200 bars	
	423.15	0.092	0.011	0.9937	0.3–6	200 bars	
	473.15	0.050	0.011	0.9800	0.3–6	200 bars	
							
	273.15	0.175	0.022	0.9934	0.3–6	400 bars	Holmes and Mesmer [27]
	298.15	0.168	0.019	0.9945	0.3–6	400 bars	
	323.15	0.158	0.017	0.9954	0.3–6	400 bars	
	373.15	0.130	0.013	0.9958	0.3–6	400 bars	
	423.15	0.096	0.011	0.9943	0.3–6	400 bars	
	473.15	0.055	0.011	0.9834	0.3–6	400 bars	

ε(Sr^2+^, Cl^-^)	273.15	0.129	0.024	0.9850	0.3–6	P_SAT_	Holmes and Mesmer [27]
	298.15	0.134	0.020	0.9902	0.3–6	P_SAT_	
	323.15	0.131	0.017	0.9926	0.3–6	P_SAT_	
	373.15	0.107	0.013	0.9935	0.3–6	P_SAT_	
	423.15	0.069	0.012	0.9878	0.3–6	P_SAT_	
	473.15	(0.028)^B^	0.012	0.9408	0.3–6	P_SAT_	
							
	273.15	0.138	0.023	0.9881	0.3–6	200 bars	Holmes and Mesmer [27]
	298.15	0.142	0.019	0.9922	0.3–6	200 bars	
	323.15	0.139	0.016	0.9942	0.3–6	200 bars	
	373.15	0.115	0.012	0.9953	0.3–6	200 bars	
	423.15	0.078	0.010	0.9925	0.3–6	200 bars	
	473.15	0.0388	0.0096	0.9765	0.3–6	200 bars	
							
	273.15	0.147	0.022	0.9904	0.3–6	400 bars	Holmes and Mesmer [27]
	298.15	0.150	0.018	0.9937	0.3–6	400 bars	
	323.15	0.146	0.015	0.9954	0.3–6	400 bars	
	373.15	0.1231	0.011	0.9965	0.3–6	400 bars	
	423.15	0.0876	0.0093	0.9953	0.3–6	400 bars	
	473.15	0.0507	0.0078	0.9902	0.3–6	400 bars	

ε(Ba^2+^, Cl^-^)	273.15	0.0645	0.0058	0.9954	0.3–9	P_SAT_	Holmes and Mesmer [27]
	298.15	0.0758	0.0048	0.9978	0.3–9	P_SAT_	
	323.15	0.0737	0.0042	0.9977	0.3–9	P_SAT_	
	373.15	0.0542	0.0034	0.9971	0.3–9	P_SAT_	
	423.15	0.0250	0.0030	0.9907	0.3–9	P_SAT_	
							
	273.15	0.0694	0.0054	0.9966	0.3–9	200 bars	Holmes and Mesmer [27]
	298.15	0.0795	0.0043	0.9984	0.3–9	200 bars	
	323.15	0.0772	0.0037	0.9987	0.3–9	200 bars	
	373.15	0.0580	0.0029	0.9986	0.3–9	200 bars	
	423.15	0.0299	0.0023	0.9966	0.3–9	200 bars	
	273.15	0.0740	0.0051	0.9974	0.3–9	400 bars	Holmes and Mesmer 27
	298.15	0.0826	0.0040	0.9987	0.3–9	400 bars	
	323.15	0.0803	0.0032	0.9991	0.3–9	400 bars	
	373.15	0.0618	0.0025	0.9990	0.3–9	400 bars	
	423.15	0.0349	0.0018	0.9984	0.3–9	400 bars	

ε(Sm^3+^, Cl^-^)	278.15	0.237	0.016	0.9934	0.51–6.0	1 bar	Roy et al. [33]
	288.15	0.228	0.015	0.9931	0.51–6.0	1 bar	
	298.15	0.211	0.017	0.9902	0.51–6.0	1 bar	
	308.15	0.207	0.014	0.9926	0.51–6.0	1 bar	
	318.15	0.194	0.014	0.9919	0.51–6.0	1 bar	
	328.15	0.180	0.014	0.9907	0.51–6.0	1 bar	
	338.15	0.167	0.015	0.9874	0.51–6.0	1 bar	

ε(Eu^3+^, Cl^-^)	278.15	0.223	0.012	0.9953	0.51–6.0	1 bar	Roy et al. [34]
	288.15	0.225	0.013	0.9945	0.51–6.0	1 bar	
	298.15	0.224	0.015	0.9934	0.51–6.0	1 bar	
	308.15	0.221	0.015	0.9927	0.51–6.0	1 bar	
	318.15	0.219	0.017	0.9908	0.51–6.0	1 bar	
	328.15	0.213	0.018	0.9889	0.51–6.0	1 bar	
	338.15	0.206	0.019	0.9867	0.51–6.0	1 bar	

ε(Gd^3+^, Cl^-^)	278.15	0.236	0.014	0.9944	0.51–6.0	1 bar	Roy et al. [28]
	288.15	0.233	0.015	0.9939	0.51–6.0	1 bar	
	298.15	0.228	0.015	0.9932	0.51–6.0	1 bar	
	308.15	0.222	0.015	0.9926	0.51–6.0	1 bar	
	318.15	0.214	0.016	0.9914	0.51–6.0	1 bar	
	328.15	0.205	0.017	0.9896	0.51–6.0	1 bar	
	338.15	0.195	0.017	0.9883	0.51–6.0	1 bar	

ε(GdAc^2+^, Cl^-^)	298.15	0.12	0.03	0.9960	0.1–2.0	1 bar	Ding and Wood [36]
	313.15	0.10	0.05	0.9710	0.1–2.0	1 bar	
	323.15	0.17	0.03	0.9580	0.1–2.0	1 bar	
	333.15	0.13	0.03	0.8794	0.1–2.0	1 bar	

ε(Li^+^, OH^-^)	273.15	(-0.03)	0.04	0.8826	0.1 to 2.0	P_SAT_	Holmes and Mesmer [35]
	298.15	(-0.06)	0.03	0.8666	0.1 to 2.0	P_SAT_	Robinson and Stokes [22], Holmes and Mesmer [35]
	323.15	(-0.04)	0.03	0.9138	0.1 to 3.0	P_SAT_	
	373.15	-0.0443	0.0088	0.9435	0.1 to 5.0	P_SAT_	
	423.15	-0.07	0.01	0.9571	0.1 to 5.0	P_SAT_	
	473.15	-0.10	0.02	0.9563	0.1 to 5.0	P_SAT_	

ε(Na^+^, OH^-^)	273.15	0.0536	0.0096	0.9541	0.1 to 5.0	P_SAT_	Holmes and Mesmer [35]
	298.15	0.0573	0.0061	0.9832		P_SAT_	
	323.15	0.0546	0.0042	0.9912		P_SAT_	
	373.15	0.0392	0.0030	0.9911		P_SAT_	
	423.15	0.0189	0.0027	0.9449		P_SAT_	
	473.15	(-0.02)	0.03	0.9130		P_SAT_	

ε(K^+^, OH^-^)	273.15	0.1017	0.0055	0.9956	0.1 to 5.0	P_SAT_	Holmes and Mesmer [35]
	298.15	0.0994	0.0039	0.9978		P_SAT_	
	323.15	0.0859	0.0029	0.9983		P_SAT_	
	373.15	0.0550	0.0029	0.9959		P_SAT_	
	423.15	0.0331	0.0039	0.9806		P_SAT_	
	473.15	(0.0189)	0.0065	0.9115		P_SAT_	

ε(Cs^+^, OH^-^)	273.15	0.1064	0.0065	0.9944	0.1 to 5.0	P_SAT_	Holmes and Mesmer [35]
	298.15	0.1061	0.0049	0.9968		P_SAT_	
	323.15	0.1015	0.0039	0.9978		P_SAT_	
	373.15	0.0839	0.0039	0.9968		P_SAT_	
	423.15	0.0615	0.0054	0.9885		P_SAT_	
	473.15	0.0435	0.0077	0.9745		P_SAT_	

ε(Sr^2+^, OH^-^)	298.15	-0.11	0.02	0.9138	0.2 to 4.0	1 bar	Johnston and Grove [40]

ε(Ba^2+^, OH^-^)	298.15	-0.10	0.01	0.8881	0.1 to 4.9	1 bar	Johnston and Grove [40]

ε(Na^+^, Ac^-^)	273.15	0.0755	0.0027	0.9999	0.1 to 2.0	1 bar	Partanen and Covington [39]
	283.15	0.0593	0.0030	0.9999	0.1 to 2.0	1 bar	Partanen and Covington [39]
	298.15	0.0399	0.0060	0.9952	0.1 to 5.0	1 bar	Kiss and Urmánczy [37]; Mesmer et al. [38]; Ding and Wood [36]; Partanen and Covington [39]
	313.15	0.03	0.02	0.9816	0.1 to 2.0	1 bar	Ding and Wood (2002) 36; Partanen and Covington [39]
	323.15	0.0304	0.0077	0.9917	0.1 to 5.0	1 bar	Mesmer et al. [38]; Ding and Wood [36]; Partanen and Covington [39]
	348.15	0.0275	0.0068	0.9980	0.1 to 5.0	1 bar	Mesmer et al. [38]
	373.15	0.0155	0.0059	0.9977	0.1 to 5.0	P_SAT_	Mesmer et al. [38]
	398.15	0.003	0.006	0.9962	0.1 to 5.0	P_SAT_	Mesmer et al. [38]
	423.15	-0.007	0.006	0.9876	0.1 to 5.0	P_SAT_	Mesmer et al. [38]
	448.15	-0.0157	0.0089	0.9643	0.1 to 5.0	P_SAT_	Mesmer et al. [38]

ε(Ca^2+^, Ac^-^)	298.15	-0.04	0.02	0.9619	0.2 to 4.9	1 bar	Johnston and Grove [40]

^A ^P_SAT _: Saturated vapor pressure; ^B ^Values in parentheses are considered to be less precise because of lower linearity of fitting or larger 2σ.

Interaction coefficients of ε(Na^+^, Cl^-^), from 0°C to 200°C at P_SAT _are evaluated from the experimental data of Gibbard et al. [25]

Interaction coefficients of ε(Mg^2+^, Cl^-^), ε(Ca^2+^, Cl^-^), ε(Sr^2+^, Cl^-^), and ε(Ba^2+^, Cl^-^) are evaluated from isopiestic studies of Holmes et al. [25] and Holmes and Mesmer [27]. Three sets of interaction coefficients of ε(Mg^2+^, Cl^-^), ε(Ca^2+^, Cl^-^), ε(Sr^2+^, Cl^-^), and ε(Ba^2+^, Cl^-^) are evaluated at three constant pressures (i.e., P_SAT_, 200 bars, and 400 bars). Similarly to ε(H^+^, Cl^-^), there is a systematic increase in interaction coefficient with pressure, although such increase is within the uncertainty of evaluation.

Roy et al. [28] experimentally determined activity coefficients of the HCl + GdCl_3 _+ H_2_O system up to 55°C by measuring electromotive force at ionic strength up to 2.0 m. Based upon the enthalpy [29] and heat capacity [30] data of GdCl_3_, they derive temperature dependence relations for the Pitzer parameters of GdCl_3 _to 55°C. In the present study, mean activity coefficients of GdCl_3 _solutions with concentrations of GdCl_3 _from 0.1 m to 1.0 m up to 65°C at 1 bar are reproduced according the temperature dependence relations of the Pitzer parameters from Roy et al. [28]. Results above 55°C to 65°C are generated by extrapolation of the temperature dependence relations of Roy et al. [28] to 65°C. Reproduction of mean activity coefficients of GdCl_3 _follows the equations defined by Pitzer and Mayorga [31] (their equations (3), (6), (9) and (11)), and the necessary Pitzer-Debye-Hückel slopes for the osmotic coefficients (A_ϕ_) at temperatures of interest and at 1 atm. are taken from Ananthaswamy and Atkinson [32]. Then, the interaction coefficients of ε(Gd^3+^, Cl^-^) up to 65°C are evaluated from the mean activity coefficients of GdCl_3_.

Similarly, Roy et al. [33] experimentally determined activity coefficients of the HCl + SmCl_3 _+ H_2_O system up to 55°C by measuring electromotive force at ionic strength up to 3.0 m. They estimated temperature dependence relations for the Pitzer parameters of SmCl_3 _to 55°C by using the enthalpy [29] and heat capacity [30] data of SmCl_3_. In this study, mean activity coefficients of SmCl_3 _solutions with concentrations of SmCl_3 _from 0.1 m to 1.0 m up to 65°C at 1 bar are reproduced according the temperature dependence relations of the Pitzer parameters from Roy et al. [33]. Then, the interaction coefficients of ε(Sm^3+^, Cl^-^) up to 65°C are evaluated from the mean activity coefficients of SmCl_3 _solutions.

In a similar study, Roy et al. [34] investigated activity coefficients of the HCl + EuCl_3 _+ H_2_O system up to 55°C by measuring electromotive force at ionic strength up to 2.0 m. They derived temperature dependence relations for the Pitzer parameters of EuCl_3 _to 55°C according to the enthalpy [29] and heat capacity [30] data of EuCl_3_. In this study, mean activity coefficients of EuCl_3 _solutions with concentrations of EuCl_3 _from 0.1 m to 1.0 m up to 65°C at 1 bar are reproduced by using the temperature dependence relations of the Pitzer parameters from Roy et al. [34]. Then, the interaction coefficients of ε(Eu^3+^, Cl^-^) up to 65°C are evaluated from the mean activity coefficients of EuCl_3 _solutions.

Holmes and Mesmer [35] presented mean activity coefficients for LiOH, NaOH, KOH, and CsOH solutions at ionic strengths up to 5.0 m based on isopiestic studies at P_SAT_. Accordingly, interaction coefficients of ε(Li^+^, OH^-^), ε(Na^+^, OH^-^), ε(K^+^, OH^-^), and ε(Cs^+^, OH^-^) are evaluated from the mean activity coefficients of Holmes and Mesmer [35].

### Interaction coefficients derived from equilibrium quotients with ionic strength dependence

Ding and Wood [36] determined the stability quotients of acetate complexes of La^3+^, Nd^3+^, Gd^3+ ^and Yb^3+ ^at 25–70°C and 1 bar in NaCl media by potentiometric titration. The stability quotient can be expressed for the following general reaction:

Ln^3+ ^+ Ac^- ^= LnAc^2+ ^    (4)

As the interaction coefficient of ε(Gd^3+^, Cl^-^) is evaluated up to 65°C in the above section, the interaction coefficient of ε(GdAc^2+^, Cl^-^) can be evaluated from their stability quotients for GdAc^2+ ^up to 60°C. The interaction coefficient of ε(GdAc^2+^, Cl^-^) at 70°C is not evaluated from their stability quotients because of poor linearity with ionic strength at that temperature.

For Reaction (4) regarding the formation of GdAc^2+ ^in NaCl media, we have:

log K°_(4) _= log Q_(4) _+ 6D + Δε(Eq. 4) I_m _    (5)

Rearranging, we have

log Q_(4) _+ 6D = log K°_(4) _- Δε(Eq. 4) I_m _    (6)

where log K°_(4) _is the stability constant at infinite dilution, and log Q_(4) _is the stability quotient at certain ionic strength, I_m _is the ionic strength of NaCl solutions on the molal scale, and Δε(Eq. 4) is given by the following expression according to the stoichiometry of Eq. (4):

Δε(Eq. 4) I_m _= ε(GdAc^2+^, Cl^-^) I_m _- ε(Gd^3+^, Cl^-^) I_m _- ε(Na^+^, Ac^-^) I_m _    (7)

According to Eq. (6), Δε(Eq. 4) can be obtained by plotting log Q_(4) _+ 6D versus I_m_, and Δε(Eq. 4) obtained in this manner are tabulated in Table 2. However, in order to derive ε(GdAc^2+^, Cl^-^) from Δε(Eq. 4) according to Eq. (7), ε(Na^+^, Ac^-^) must be known.

**Table 2:** Δε(Eq. 4) evaluated from formation quotients of GdAc^2+ ^of Ding and Wood [36]

Temperature, K	Δε(Eq. 4)	2σ	Linearity of Fitting (Square of Linear Correlation Coefficient, R^2^)
298.15	0.228	0.022	0.9960
313.15	0.158	0.036	0.9710
323.15	0.074	0.016	0.9580
333.15	(0.04)	0.02	0.8794

To achieve this, the interaction coefficients of ε(Na^+^, Ac^-^) at temperatures up to 175°C are evaluated from dissociation quotients of HAc in NaCl media assuming unity for the activity coefficient of HAc:

HAc = H^+ ^+ Ac^- ^(8)

Similar to Eq. (6), we have

log Q_(8) _- 2D = log K°_(8) _- Δε(Eq. 8) I_m _    (9)

where Δε(Eq. 8) is given by

Δε(Eq. 8) = [ε(H^+^, Cl^-^) + ε(Na^+^, Ac^-^)]     (10)

Therefore, when Δε(Eq. 8) are evaluated according to Eq. (9), ε(Na^+^, Ac^-^) then can be derived in combination with ε(H^+^, Cl^-^) evaluated in this study.

In estimation of Δε(Eq. 8), dissociation quotients of HAc in NaCl media determined by Kiss and Urmáncy [37], Mesmer et al. [38], Ding and Wood [36] and Partanen and Covington [39] are utilized, and Δε(Eq. 8) are tabulated in Table 3

**Table 3:** Δε(Eq. 8) evaluated from dissociation quotients of acetic acid (log Q) in NaCl solutions

Temp., K	Δε(Eq. 8)	I range, m	2σ	Linearity of Fitting (Square of Linear Correlation Coefficient, R^2^)	Data Sources for Evaluation
273.15	0.2047	0.1–2.0	0.0015	0.9999	Partanen and Covington [39]
283.15	0.1903	0.1–2.0	0.0020	0.9999	Partanen and Covington [39]
298.15	0.1704	0.1–5.0	0.0056	0.9952	Kiss and Urmánczy [37]; Mesmer et al. [38]; Ding and Wood [36]; Partanen and Covington [39]
313.15	0.148	0.1–2.0	0.028	0.9816	Ding and Wood [36]; Partanen and Covington [39]
323.15	0.1405	0.1–5.0	0.0074	0.9917	Mesmer et al. [38]; Ding and Wood [36]; Partanen and Covington [39]
348.15	0.1232	0.1–5.0	0.0064	0.9980	Mesmer et al. [38]
373.15	0.0995	0.1–5.0	0.0055	0.9977	Mesmer et al. [38]
398.15	0.0753	0.1–5.0	0.0053	0.9962	Mesmer et al. [38]
423.15	0.0506	0.1–5.0	0.0065	0.9876	Mesmer et al. [38]
448.15	0.0283	0.1–5.0	0.0085	0.9643	Mesmer et al. [38]

Johnston and Grove [40] studied the solubility of portlandite in various media at 25°C. According to the solubility product quotients of portlandite in NaAc, SrCl_2 _and BaCl_2 _regarding the following reaction:

Ca(OH)_2 _(s) = Ca^2+ ^+ 2OH^- ^    (11)

the interaction coefficients of ε(Ca^2+^, Ac^-^), ε(Sr^2+^, OH^-^), and ε(Ba^2+^, OH^-^) at 25°C are also estimated from the experimental data of Johnston and Grove [40]. These interaction coefficients are derived from Δε(Eq. 11) listed in Table 4 in conjunction with ε(Na^+^, OH^-^) or ε(Ca^2+^, Cl^-^) at 25°C estimated in this study. These interaction coefficients, ε(Ca^2+^, Ac^-^), ε(Sr^2+^, OH^-^), and ε(Ba^2+^, OH^-^), are not covered in Ciavatta [12,13], and therefore are not compiled in Grenthe et al. [14] nor in Guillaumont et al. [15].

**Table 4:** Δε(Eq. 11) estimated from solubility product quotients of portlandite in various media at 25°C

Medium	Δε(Eq. 11) (± 2σ)	Ionic Strength Range, m	Linearity of Fitting (Square of Linear Correlation Coefficient, R^2^)	Experimental Data Source for Estimation
NaAc	0.07 ± 0.02	0.2–4.9	0.9619	Johnston and Grove [40]
SrCl_2_	-0.06 ± 0.02	0.2–4.0	0.9138	
BaCl_2_	-0.035 ± 0.008	0.1–4.9	0.8881	

Seward [41] determined the solubilities of AgCl (s) (cerargyrite) in NaCl solutions of up to 3.0 m. For the solubility product constant, K°_s0_, the reaction can be expressed as

AgCl (s) = Ag^+ ^+ Cl^- ^    (12)

In the study of Seward [41], equilibrium constants such as K°_s0 _were extrapolated to infinite dilution by using the B dot equation [9], and equilibrium quotients (conditional equilibrium constants) were not presented. In this study, respective equilibrium quotients are recalculated according to the B dot equation. For instance, Q_s0 _are recalculated as



In the work of Seward [41], log  was calculated by using the B dot equation. Log  was calculated from the stoichiometric mean activity coefficient of NaCl from Liu and Lindsay [42]:



where α is the degree of dissociation of the NaCl° ion pair and is defined as:



In the work of Seward [41], unity is assumed for γ_NaClo_, and a temperature dependence expression was given for dissociation constants of NaCl° (K_d_).

Accordingly, for Reaction (12) in NaCl media, we have:

log Q_s0(12) _- 2D = log K°_s0(12) _- Δε(Eq. 12) I_m _    (16)

where Δε(Eq. 12) is given by

Δε(Eq. 12) = [ε(Ag^+^, Cl^-^) + ε(Na^+^, Cl^-^)]     (17)

The estimated Δε(Eq. 12) are listed in Table 5. Therefore, based upon ε(Na^+^, Cl^-^) evaluated before, ε(Ag^+^, Cl^-^) can be derived from Δε(Eq. 12), and they are listed in Table 1.

**Table 5:** Δε(Eq. 12) evaluated from solubility quotients (logQ_s0_) of AgCl (s) in NaCl solutions

Temp., K	Δε(Eq.12)	I range, m	2σ	Linearity of Fitting (Square of Linear Correlation Coefficient, R^2^)	Data Sources for Evaluation
373.15	0.127	0.5–3.0	0.014	0.9842	Seward [41]
423.15	0.144	0.5–3.0	0.019	0.9825	
470.15	0.152	0.5–3.0	0.019	0.9840	

Similarly, for the cumulative equilibrium quotient, Q_2_, we have,

Ag^+ ^+ 2Cl^- ^= AgCl_2 _^- ^(18)

Accordingly, we have the following expression:

log Q_2(18) _+ 2D = log β°_2(18) _- Δε I_m _    (19)

where Δε(Eq. 18) is defined by

Δε(Eq. 18) = [ε(Na^+^, AgCl_2 _^-^) - ε(Ag^+^, Cl^-^) - 2ε(Na^+^, Cl^-^)]     (20)

The evaluated Δε(Eq. 18) are listed in Table 6. According to ε(Na^+^, Cl^-^) and ε(Ag^+^, Cl^-^) evaluated above, ε(Na^+^, AgCl_2 _^-^) can be derived from Δε(Eq. 18), and they are tabulated in Table 1.

**Table 6:** Δε(Eq. 18) evaluated from the cumulative equilibrium quotients of AgCl (log Q_2_) in NaCl

Temp., K	Δε(Eq.18)	I range, m	2σ	Linearity of Fitting (Square of Linear Correlation Coefficient, R^2^)	Data Sources for Evaluation
373.15	-0.194	0.5–3.0	0.023	0.9821	Seward [41]
423.15	-0.227	0.5–3.0	0.032	0.9807	
470.15	-0.243	0.5–3.0	0.032	0.9832	

**Figure 2:**
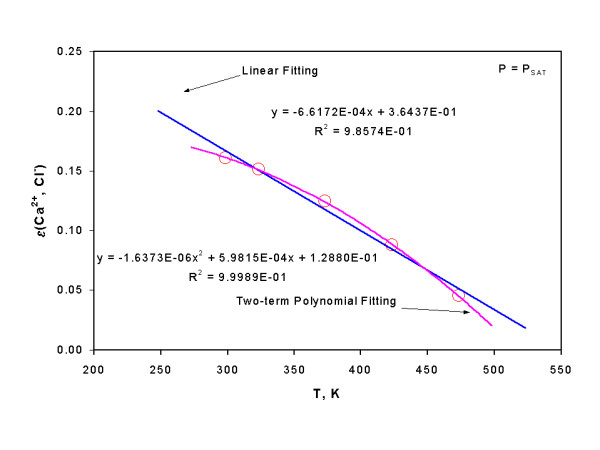
A plot showing ε(Ca^2+^, Cl^-^) as a function of temperature at saturated vapor pressures.

Based upon the interaction coefficients estimated at various temperatures, their respective temperature dependence expressions are presented in Table 7. While two-term polynomial fittings can almost exactly reproduce interaction coefficients at various temperatures, linear fittings for some interaction coefficients can also reproduce interaction coefficients at various temperatures within uncertainty of 2σ as exemplified by the case for ε(Ca^2+^, Cl^-^) at various temperatures (Figure 2). Although both linear and two-term polynomial expressions are given for some interaction coefficients, it is recommended to use two-term polynomial expressions for interpolations. Linear relations for some interaction coefficients might be used for extrapolations over a limited range of temperatures beyond the temperatures at which they are evaluated (say, over a limited range of ~25°C). For instance, as ε(Na^+^, AgCl_2 _^-^) is evaluated at 100°C–197°C, it might be possible to extrapolate the interaction coefficient to 75°C by using its linear expression.

**Table 7:** Temperature dependence expressions for interaction coefficients involving Cl^-^, OH^- ^and Ac^- ^derived from this study

Interaction Coefficients	Temperature Dependence Expressions, T in K	Average 2σ	Ionic Strength Range, m	Temperature Range and Pressure
ε(H^+^, Cl^-^)	Linear:ε = 2.7890 × 10^-1 ^- 5.2237 × 10^-4 ^T (R^2^= 0.9950)	0.0019	0.1–6.0	298.15–473.15 K at P_SAT_
	Polynomial:ε = 3.8988 × 10^-1 ^- 1.0783 × 10^-3^T -6.9000 × 10^-7 ^T^2 ^(R^2 ^= 0.9968)			
ε(H^+^, Cl^-^)	Linear: ε = 2.5259 × 10^-1 ^- 4.4384 × 10^-4 ^T (R^2^= 0.9985)	0.0010	0.1–2.0	273.15–473.15 K at 400 bars
	Polynomial:ε = 2.1154 × 10^-1 ^- 2.0587 × 10^-4^T -3.3783 × 10^-7 ^T^2 ^(R^2 ^= 0.9998)			
ε(Na^+^, Cl^-^)	ε = -4.1341 × 10^-2 ^+ 5.8237 × 10^-4 ^T - 9.5405 × 10^-7 ^T^2 ^(R^2 ^= 0.9974)	0.0007	0.5–6.0	298.15–473.15 K at P_SAT_
ε(Na^+^, AgCl_2 _^-^)	Linear: ε = 1.7132 × 10^-1 ^- 5.1636 × 10^-4 ^T (R^2 ^= 0.9906)	0.03	0.5–3.0	373.15–473.15 K at P_SAT_
	Polynomial:ε = 4.8789 × 10^-1 ^- 2.0323 × 10^-3^T -1.7986 × 10^-6 ^T^2 ^(R^2 ^= 1.0000)			
ε(Ag^+^, Cl^-^)	ε = 0.11	0.02	0.5–3.0	373.15–473.15 K at P_SAT_
ε(Mg^2+^, Cl^-^)	Linear:ε = 4.4121 × 10^-1 ^- 7.7283 × 10^-4 ^T (R^2^= 0.9995)	0.030	0.3–6.0	273.15–473.15 K at P_SAT_
	Polynomial:ε = 4.4442 × 10^-1 ^- 7.9072 × 10^-4^T +2.4016 × 10^-8 ^T^2 ^(R^2 ^= 0.9995)			
ε(Mg^2+^, Cl^-^)	Linear:ε = 4.3772 × 10^-1 ^- 7.5556 × 10^-4 ^T (R^2^= 0.9994)	0.031	0.3–6.0	273.15–473.15 K at 200 bars
	Polynomial:ε = 3.9380 × 10^-1 ^- 5.1076 × 10^-4^T -3.2860 × 10^-7 ^T^2 ^(R^2 ^= 0.9999)			
ε(Mg^2+^, Cl^-^)	Linear:ε = 4.3685 × 10^-1 ^- 7.3773 × 10^-4 ^T (R^2^= 0.9999)	0.029	0.3–6.0	273.15–473.15 K at 400 bars
	Polynomial:ε = 4.2439 × 10^-1 ^- 6.6827 × 10^-4^T -9.3235 × 10^-8 ^T^2 ^(R^2 ^= 0.9999)			
ε(Ca^2+^, Cl^-^)	Linear:ε = 3.6437 × 10^-1 ^- 6.6172 × 10^-4 ^T (R^2^= 0.9857)	0.030	0.3–6.0	298.15–473.15 K at P_SAT_
	Polynomial:ε = 1.2880 × 10^-1 ^+ 5.9815 × 10^-4^T -1.6373 × 10^-6 ^T^2 ^(R^2 ^= 0.9999)			
ε(Ca^2+^, Cl^-^)	Linear:ε = 3.6445 × 10^-1 ^- 6.5439 × 10^-4 ^T (R^2^= 0.9919)	0.020	0.3–6.0	298.15–473.15 K at 200 bars
	Polynomial:ε = 1.9626 × 10^-1 ^+ 2.4509 × 10^-4^T -1.1690 × 10^-6 ^T^2 ^(R^2 ^= 0.9993)			
ε(Ca^2+^, Cl^-^)	Linear:ε = 3.6440 × 10^-1 ^- 6.4277 × 10^-4 ^T (R^2^= 0.9891)	0.020	0.3–6.0	298.15–473.15 K at 400 bars
	Polynomial:ε = 1.6451 × 10^-1 ^+ 4.2626 × 10^-4^T -1.3893 × 10^-6 ^T^2 ^(R^2 ^= 0.9999)			
ε(Sr^2+^, Cl^-^)	Linear:ε = 3.2535 × 10^-1 ^- 6.1153 × 10^-4 ^T (R^2^= 0.9716)	0.018	0.3–6.0	298.15–473.15 K at P_SAT_
	Polynomial:ε = 3.1779 × 10^-2 ^+ 9.5849 × 10^-4^T -2.0404 × 10^-6 ^T^2 ^(R^2 ^= 0.9970)			
ε(Sr^2+^, Cl^-^)	Linear:ε = 3.2888 × 10^-1 ^- 5.9746 × 10^-4 ^T (R^2^= 0.9734)	0.016	0.3–6.0	298.15–473.15 K at P_SAT_
	Polynomial:ε = 5.2527 × 10^-2 ^+ 8.8048 × 10^-4^T -1.9207 × 10^-6 ^T^2 ^(R^2 ^= 0.9970)			
ε(Sr^2+^, Cl^-^)	Linear:ε = 3.2226 × 10^-1 ^- 5.5384 × 10^-4 ^T (R^2^= 0.9801)	0.014	0.3–6.0	298.15–473.15 K at P_SAT_
	Polynomial:ε = 1.2297 × 10^-1 ^+ 5.1201 × 10^-4^T -1.3852 × 10^-6 ^T^2 ^(R^2 ^= 0.9945)			
ε(Ba^2+^, Cl^-^)	Linear:ε = 2.0474 × 10^-1 ^- 4.1667 × 10^-4 ^T (R^2^= 0.9584)	0.014	0.3–9.0	298.15–423.15 K at P_SAT_
	Polynomial:ε = -1.1827 × 10^-1 ^+ 1.4053 × 10^-3^T -2.5230 × 10^-6 ^T^2 ^(R^2 ^= 0.9985)			
ε(Ba^2+^, Cl^-^)	Linear:ε = 2.0491 × 10^-1 ^- 4.0566 × 10^-4 ^T (R^2^= 0.9599)	0.013	0.3–9.0	298.15–423.15 K at 200 bars
	Polynomial:ε = -1.0310 × 10^-1 ^+ 1.3317 × 10^-3^T -2.4058 × 10^-6 ^T^2 ^(R^2 ^= 0.9985)			
ε(Ba^2+^, Cl^-^)	Linear:ε = 2.0325 × 10^-1 ^- 3.9039 × 10^-4 ^T (R^2^= 0.9621)	0.030	0.3–9.0	298.15–423.15 K at 200 bars
	Polynomial:ε = -8.3861 × 10^-2 ^+ 1.2291 × 10^-3^T -2.2426 × 10^-6 ^T^2 ^(R^2 ^= 0.9984)			
ε(Sm^3+^, Cl^-^)	Linear:ε = 5.5890 × 10^-1 ^- 1.1536 × 10^-3 ^T (R^2^= 0.9895)	0.016	0.51–6.0	273.15–338.15 K at 1 bar
	Polynomial:ε = 3.0000 × 10^-1 ^+ 5.3392 × 10^-4^T -2.7381 × 10^-6 ^T^2 ^(R^2 ^= 0.9911)			
ε(Eu^3+^, Cl^-^)	ε = -4.8708 × 10^-1 ^+ 4.8872 × 10^-3 ^T -8.3929 × 10^-6 ^T^2 ^(R^2 ^= 0.9950)	0.016	0.51–6.0	273.15–338.15 K at 1 bar
ε(Gd^3+^, Cl^-^)	Linear:ε = 4.3225 × 10^-1 ^- 6.9179 × 10^-4 ^T (R^2^= 0.9681)	0.016	0.51–6.0	273.15–338.15 K at 1 bar
	Polynomial:ε = 2.5104 × 10^-1 ^+ 3.7617 × 10^-3^T -7.2262 × 10^-6 ^T^2 ^(R^2 ^= 0.9998)			
ε(GdAc^2+^, Cl^-^)	ε = 0.12	0.04	0.1–2.0	298.15–338.15 K at 1 bar
ε(Li^+^, OH^-^)	ε = 1.5802 × 10^-1 ^- 5.4117 × 10^-4 ^T (R^2 ^= 0.9974)	0.02	0.1–5.0	298.15–473.15 K at P_SAT_
ε(Na^+^, OH^-^)	ε = -1.5573 × 10^-1 ^+ 1.4279 × 10^-3 ^T - 2.4094 × 10^-6 ^T^2 ^(R^2 ^= 0.9987)	0.0092	0.1–5.0	273.15–473.15 K at P_SAT_
ε(K^+^, OH^-^)	ε = 2.6120 × 10^-1 ^- 5.4520 × 10^-4 ^T (R^2 ^= 0.9924)	0.0043	0.1–5.0	273.15–423.15 K at P_SAT_
ε(Cs^+^, OH^-^)	ε = 2.0759 × 10^-1 ^- 3.3855 × 10^-4 ^T (R^2 ^= 0.9700)	0.0054	0.1–5.0	273.15–423.15 K at P_SAT_
ε(Na^+^, Ac^-^)	Linear:ε = 1.4784 × 10^-1 ^- 3.6016 × 10^-4 ^T (R^2 ^= 0.9928)	0.0070	0.1–5.0	298.15–448.15 K at P_SAT_
	Polynomial:ε = 3.7001 × 10^-1 ^+ 2.5583 × 10^-4^T -8.3336 × 10^-7 ^T^2 ^(R^2 ^= 0.9933)			

## Model verification and applications

As the intent of this study is to provide a framework to treat medium dependence of equilibrium constants at elevated temperatures by using the SIT model, consequently, the model verification is focused on testing whether interaction coefficients recommended by this study can provide reasonable accuracy in reproduction of medium effects on equilibrium constants. To achieve this goal, experimentally determined mean activity coefficients and equilibrium quotients at various ionic strengths are compared with respective values predicted by using the interaction coefficients estimated by this study. The strategy in the model verification is to use experimental data from independent studies, which are not considered in estimation of interaction coefficients.

### Mean activity coefficient data

In comparison of predicted mean activity coefficients with experimental values, differences are given in sigma values (σ). Similarly to Christov and Moller [43], sigma is defined as follows for mean activity coefficients:



where *x*(i)_exp _is the value of the *i*th experimental data point of the quantity *x*, *x*(i)_pred _is the corresponding predicted value of the quantity *x*, and *n *is the number of points in the data set.

In Table 8, the interaction coefficients estimated in this study are tested against independent experimental data on mean activity coefficients. The bulk of experimental mean activity coefficient data prior to 1989 are those selected by Lobo [44] and Lobo and Quaresma [45]. The σ values listed in Table 8 indicate the expected accuracy when these interaction coefficients are used. For some interaction coefficients, especially those involving complexes, because experimental data on mean activity coefficient are lacking, they are tested against equilibrium quotients (see below).

**Table 8:** Comparison of mean activity coefficients predicted by using SIT with experimental values

System	Experimental Data Sets*	Sigma Value σ
HCl-H_2_O	Robinson and Harned [46]: m_HCl _= 0.0001–4.0, T = 0–60°C	0.0194
	Bates and Bower [47]: m_HCl _= 0.001–1.0, T = 0–90°C	0.0121
	Greeley et al. [23]: m_HCl _= 0.001–1.0, T = 60–200°C	0.0081
	Roy et al. [48]: m_HCl _= 0.005–4.0, T = 0–45°C	0.0171
		
NaCl-H_2_O	Robinson and Harned [46]: m_NaCl _= 0.1–4.0, T = 0–100°C	0.0295
	Liu and Lindsay [42]: m_NaCl _= 0.05–6.0, T = 75–200°C	0.0167
	Busey et al. [49]: m_NaCl _= 0.1–5.0, T = 100–200°C	0.0135
		
HCl-MgCl_2_-H_2_O	White et al. [50]: m_HCl _= 0.01–0.7848, m_MgCl2 _= 0.0033–0.2616; T = 0–45°C	γ_HCl_: 0.0114
		
HCl-BaCl_2_-H_2_O	Roy et al. [48]: m_HCl _= 0.0025–2.0, m_BaCl2 _= 0.000833–0.667; T = 0–45°C	γ_HCl_: 0.0391 γ_BaCl2_: 0.0180
		
MgCl_2_-H_2_O	Pan [51]: m_MgCl2 _= 0.0001–0.1, T = 25°C	0.0011
	Goldberg and Nuttall [52]: m_MgCl2 _= 0.001–2.0, T = 25°C	0.0164
	Rard and Miller [53]: m_MgCl2 _= 0.1––2.0, T = 25°C	0.0348
	Wang et al. [54]: m_MgCl2 _= 0.001–2.0, T = 100–200°C (theoretical fit)	0.0111
	El Guendouzi et al. [55]: m_MgCl2 _= 0.2–2.0, T = 25°C	0.0322
		
CaCl_2_-H_2_O	McLeod and Gordon [56]: m_CaCl2 _= 0.0016–0.0784, T = 15°C–35°C	0.0006
	Robinson [57]: m_CaCl2 _= 0.1–2.2, T = 25°C	0.025
	Stokes [58]: m_CaCl2 _= 0.1–3.0, T = 25°C	0.0200
	Shedlovsky [59]: m_CaCl2 _= 0.001–0.1, T = 25°C	0.0039
	Robinson and Stokes [22]: m_CaCl2 _= 0.1–3.0, T = 25°C	0.0200
	Staples and Nuttall [60]: m_CaCl2 _= 0.001–3.0, T = 25°C	0.0145
	Rard and Clegg [61]: m_CaCl2 _= 0.001–3.0, T = 25°C	0.0159
	El Guendouzi et al. [55]: m_CaCl2 _= 0.2–3.0, T = 25°C	0.0205
	Gruszkiewicz and Simonson [62]: m_CaCl2 _= 0.01–3.0, T = 50°C–200°C	0.0110
		
SrCl_2_-H_2_O	Harned and Åkerlöf [63]: m_SrCl2 _= 0.01–1.0, T = 25°C	0.0280
	Phillips et al. [64]: m_SrCl2 _= 0.05–1.3, T = 25°C	0.0414
	Robinson and Stokes [22]: m_SrCl2 _= 0.1–3.0, T = 25°C	0.0404
	Longhi et al. [65]: m_SrCl2 _= 0.0001–0.3, T = 10°C–70°C	0.0109
	Pan [51]: m_SrCl2 _= 0.0001–0.1, T = 25°C	0.0012
	Goldberg and Nuttall [52]: m_SrCl2 _= 0.001–3.0, T = 25°C	0.0401
		
BaCl_2_-H_2_O	Tippetts and Newton [66]: m_BaCl2 _= 0.01–1.8, T = 0°C–45°C	
	Ardizzone et al. [67]: m_BaCl2 _= 0.0001–0.3, T = 10°C–70°C	
		
SmCl_3_-H_2_O	Mason [68]: m_SmCl3_= 0.05–1.0, T = 25°C	0.0218
	Robinson and Stokes [22]: m_SmCl3_= 0.1–1.0, T = 25°C	0.0334
	Spedding et al. [69]: m_SmCl3_= 0.1–1.0, T = 25°C	0.0247
		
EuCl_3_-H_2_O	Mason [68]: m_EuCl3_= 0.05–1.0, T = 25°C	0.0220
	Robinson and Stokes [22]: m_EuCl3_= 0.1–1.0, T = 25°C	0.0317
	Spedding et al. [69]: m_EuCl3_= 0.1–1.0, T = 25°C	0.0254
		
GdCl_3_-H_2_O	Spedding et al. [69]: m_EuCl3_= 0.1–1.0, T = 25°C	0.0247
		
LiOH-H_2_O	Kangro and Groeneveld [70]: m_LiOH _= 0.5–2.0, T = 25°C	0.0375
	Hamer and Wu [71]: m_LiOH _= 0.001–2.0, T = 25°C	0.0290
		
NaOH-H_2_O	Harned [72]: m_NaOH _= 0.0202–3.1, T = 25°C	0.0174
	Harned and Åkerlöf [63]: m_NaOH _= 0.0053–3.1, T = 25°C	0.0274
	Åkerlöf and Kegeles [73]: m_NaOH _= 0.1–5.0, T = 0–70°C	0.0382
	Robinson and Stokes [22]: m_NaOH _= 0.1–5.0, T = 25°C	0.0365
	Hamer and Wu [71]: m_NaOH _= 0.001–5.0, T = 25°C	0.0298
		
KOH-H_2_O	Knobel [74]: m_KOH _= 0.001–3.0, T = 25°C	0.0178
	Scatchard [75]: m_KOH _= 0.001–1.0, T = 25°C	0.0209
	Harned and Åkerlöf [63]: m_KOH _= 0.01–1.0, T = 25°C	0.0201
	Harned and Cook [76]: m_KOH _= 0.1–4.0, T = 0°C–35°C	0.0345
	Robinson and Harned [46]: m_KOH _= 0.05–4.0, T = 25°C	0.0207
	Robinson and Stokes [22]: m_KOH _= 0.1–5.0, T = 25°C	0.0422
	Hamer and Wu [71]: m_KOH _= 0.001–5.0, T = 25°C	0.0377
		
CsOH-H_2_O	Harned and Schupp [77]: m_CsOH _= 0.01016–1.3205, T = 25°C	0.0129
	Robinson and Stokes [78]: m_CsOH _= 0.1–1.0, T = 25°C	0.0171
	Robinson and Stokes [22]: m_CsOH _= 0.1–1.0, T = 25°C	0.0083
	Hamer and Wu [71]: m_CsOH _= 0.001–1.2, T = 25°C	0.0086

* Multiple experimental data sets in the same system are listed in chronological order.

### Equilibrium quotient data

Similar to the sigma value defined for comparison of mean activity coefficients, the sigma value for the difference in equilibrium quotient is defined as:



where log *Q*_exp _is the experimental equilibrium quotient at certain ionic strength (in molality) in logarithmic unit, log *Q*_pred _is the equilibrium quotient at the same strength in logarithmic unit predicted by using the SIT model.

The first example is to compare the solubility equilibrium quotients of brucite in NaCl, NaCl+MgCl_2 _and MgCl_2 _solutions with ionic strength up to 8 m at temperatures up to 200°C. The solubility equilibrium of brucite can be expressed as:

Mg(OH)_2 _(s) + 2H^+ ^= Mg^2+ ^+ 2H_2_O (l)     (23)

The expression for the equilibrium constant at infinite dilution can be written as:

log K° = log Q - 2D + ε(Mg^2+^, Cl^-^) I_m _- 2ε(H^+^, Cl^-^) I_m _+ 2 log      (24)

Rearrangement of Eq. (24) gives:

log Q = log K° + 2D - ε(Mg^2+^, Cl^-^) I_m _+ 2ε(H^+^, Cl^-^) I_m _- 2 log      (25)

Therefore, according to the interaction coefficients recommended by this study and the equilibrium constant at infinite dilution, the equilibrium quotient at certain ionic strength can be predicted.

Altmaier et al. [79] determined solubility equilibrium quotient of brucite in NaCl and MgCl_2 _solutions, and they obtained its solubility constant at infinite dilution (logK°) by extrapolation by using the Pitzer equations as 17.1 ± 0.2.

**Figure 3:**
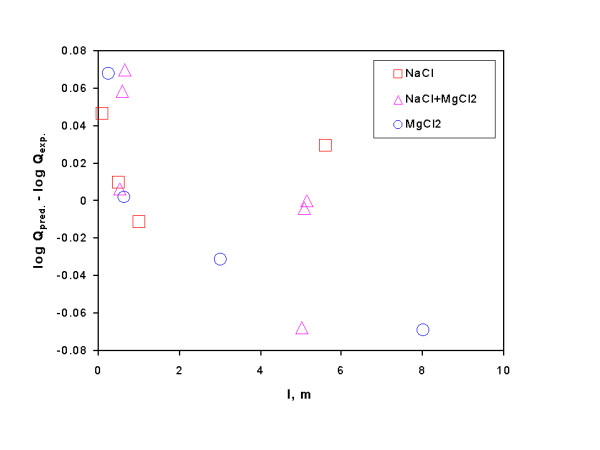
A plot showing the comparison of predicted solubility quotients of brucite with those determined by experimental studies in NaCl, NaCl+MgCl_2 _and MgCl_2 _solutions at 25°C. Experimental solubility quotients are from Altmaier et al. 79.

In Eq. (25), the activity of water in NaCl solutions at 25°C is taken from Robinson and Stokes [22] and in MgCl_2 _solutions is taken from Rard and Miller [53], and log K° (17.1 ± 0.2) is taken from Altmaier et al. [79]. It is obvious from Figure 3 that the interaction coefficients of ε(Mg^2+^, Cl^-^) and ε(H^+^, Cl^-^) evaluated by this study can reproduce within the experimental uncertainty the solubility equilibrium quotients of brucite at ionic strength up to 8.0 m in NaCl, NaCl+MgCl_2 _and MgCl_2 _solutions at 25°C. Additionally, it may be worth noting that in the extrapolation of their experimental data to infinite dilution by using the Pitzer equations, Altmaier et al. [79] employed 20 Pitzer parameters (12 binary interaction parameters and 8 ternary/mixing interaction parameters) to describe fully their experimental system when MgOH^+ ^is ignored. In contrast, only 2 parameters are needed to describe fully the experimental system by the SIT. Therefore, the SIT is mathematically simpler, and is able to describe the system with the reasonable accuracy.

**Figure 4:**
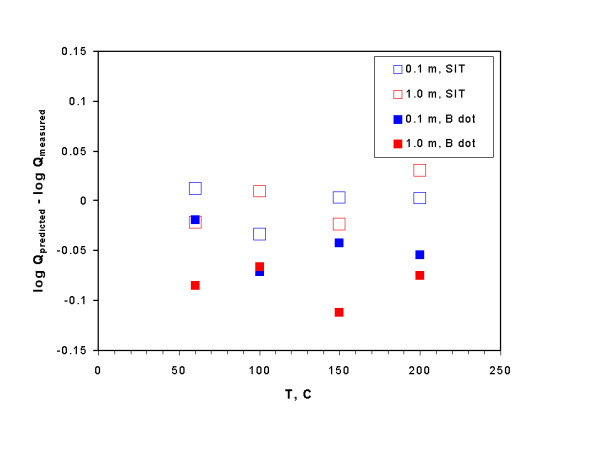
A plot showing the comparison of predicted solubility quotients of brucite with those determined by experimental studies in NaCl at elevated temperatures. Experimental solubility quotients are at 60°C, 100°C, 150°C, and 200°C are from Brown et al. 80.

Brown et al. [80] determined by potentiometric titration the solubility equilibrium quotients of brucite in 0.1 m and 1.0 m NaCl solution at 60, 100, 150, and 200°C. In calculations of equilibrium quotients, activity of water in Eq. (25) below 75°C is taken from Gibbard et al. [25], and at T ≥ 75°C is taken from Liu and Lindsay [42], and the logK° at the above temperatures are taken from Brown et al. [80]. It can be seen from Figure 4 that the predicted equilibrium quotients are in agreement with the experimental values within the experimental uncertainty, as differences are less than ± 0.04 log units, and the two standard deviations (2σ) associated with their equilibrium quotients are ± 0.04. In addition, solubility quotients of brucite are also predicted by using the B dot equation [9] for comparison (Figure 4). In using the B dot equation, the ion size parameters for Mg^2+ ^and H^+ ^are from the compilation of Wolery [10], and B dot parameters are from Helgeson [9]. It seems clear from Figure 4 that all of the differences between solubility quotients predicted by the B dot equation and those determined by the experimental studies exceed ± 0.04 log units except one data point in 0.1 m NaCl solution at 60°C, which is within the experimental uncertainty of ± 0.04 log units. Therefore, the SIT model performs better than the B dot equation does even in the ionic strength range valid for the B dot equation, although the B dot equation does have the general applicability as it does not require any specific interaction coefficients.

The second case is to compare the predicted solubility of cerargyrite, AgCl(s), in 1.0 m HCl at 100°C with the experimental solubility of Gammons and Seward [81]. The following two considerations are taken into account into choosing to compare the solubility of AgCl(s) in 1.0 m NaCl solution at 100°C. The first consideration is that that AgCl_2 _^- ^is the dominant species in 1.0 m NaCl solution at 100°C [41]. The second consideration is that the association of H^+ ^with Cl^- ^to form ion pair HCl° is insignificant at this temperature [82]. These two facts will contribute to the simplification of the comparison. It also should be mentioned that the data of Gammons and Seward [81] have not been used to derive interaction coefficients in this study. Therefore, they are independent of interaction coefficients derived from Seward [41].

The solubility of AgCl(s) as AgCl_2 _^- ^can be expressed as:

AgCl(s) + Cl^- ^= AgCl_2 _^- ^    (26)

log K°_s2 _= -3.19 ± 0.16 (2σ)

The above log K°_s2 _at infinite dilution at 100°C is obtained by combination of log K°_s0 _and log β°_2 _of Seward [41], and the uncertainty is calculated as two standard deviations (2σ) and error propagation is taken into consideration. According to Reaction (26), the relation between log K°_s2 _and log Q_s2 _can be written as:

log Q_s2 _= log K°_s2 _- ε(H^+^, AgCl_2 _^-^) + ε(H^+^, Cl^-^)     (27)

When log Q_s2 _is calculated, it is assumed that ε(H^+^, AgCl_2 _^-^) ≅ ε(Na^+^, AgCl_2 _^-^). The calculated log Q_s2 _at 1.0 m HCl is -3.08 ± 0.16 (2σ). Therefore, the predicted solubility of AgCl(s) in 1.0 m HCl at 100°C is 10^-3.08 ± 0.16 ^m (see Table 9). Notice that the uncertainty is inherited from log K°_s2_. The measured solubility of Gammons and Seward [81] in the same solution at the same temperature is 10^-2.88^m. In the above calculations, the small contributions from AgCl°, AgCl_3 _^-2 ^and AgCl_4 _^-3 ^are ignored. Accordingly, when contributions from AgCl°, AgCl_3 _^-2 ^and AgCl_4 _^-3 ^are considered, the predicted solubility of Ag(s) in 1.0 m HCl would be in satisfactory agreement with the measured solubility.

**Table 9:** Predicted solubility quotient of AgCl(s), and predicted and measured solubility of AgCl(s), in HCl solutions at 100°C

I, m_HCl_	logQ_s2_	Predicted Solubility, m	Measured Solubility, m, from Gammons and Seward [81]
1.0	-3.08 ± 0.16	10^-3.08 ± 0.16^	10^-2.88^
2.0	-2.98 ± 0.16	10^-2.68 ± 0.16^	Not measured
3.0	-2.87 ± 0.16	10^-2.40 ± 0.16^	10^-2.23^

In addition, log Q_s2 _in 3.0 m HCl at 100°C is also predicted according to Reaction (26) (Table 9). By using log Q_s2 _in 3.0 m HCl at 100°C, the solubility of AgCl(s) can be satisfactorily predicted by assuming the sole contribution from AgCl_2 _^- ^(Table 9), as the predicted solubility is 10^-2.40 ± 0.16 ^m in comparison with the measured solubility of 10^-2.23^m. Therefore, the dominance field of AgCl_2 _^- ^may well be extended to chloride concentrations of 3.0 m.

The third case is the comparison of the ionization of water in LiCl, NaCl and KCl solutions ranging from 0.1 m to 5.0 m at temperatures up to 200°C determined by various researchers with the values predicted by using ε(H^+^, Cl^-^), ε(Li^+^, OH^-^), ε(Na^+^, OH^-^) and ε(K^+^, OH^-^) derived in this study. The ionization constant of water, for example, in NaCl media, can be expressed as:

H_2_O (l) = H^+ ^+OH^- ^    (28)

log K°_w _= log Q_w _- 2D + ε(H^+^, Cl^-^) m_NaCl _+ ε(Na^+^, OH^-^) m_NaCl _- log     (29)

where log K°_w _is the ionization constant of water at infinite dilution, log Q_w _the apparent ionization constant of water at a certain ionic strength. Eq. (29) can be recast as:

log Q_w _= log K°_w _+ 2D - ε(H^+^, Cl^-^) m_NaCl _- ε(Na^+^, OH^-^) m_NaCl _+ log      (30)

The activity of water in NaCl solutions is calculated from the osmotic coefficient (ϕ) of the NaCl solutions with NaCl concentrations of interest,

log = -2 m_NaCl_ϕ/[(ln10) × 55.51]     (31)

and the osmotic coefficients of NaCl solutions are from Gibbard et al. [25] at T < 75°C, and from Liu and Lindsay [42] at T ≥ 75°C.

According to Eq. (30) and log K°_w _from Busey and Mesmer [83], the apparent dissociation quotients of water in LiCl, NaCl and KCl media with ionic strength up to 5.0 m are predicted based the interaction coefficients recommended by this study. In calculations, the osmotic coefficients of LiCl solutions at 25°C are from Robinson and Stokes [22], and the osmotic coefficients of KCl solutions are assumed to be the same as those of NaCl solutions. The predicted values are compared with the experimentally determined values (Table 10). As indicated in Table 10, although the predicted values are not exactly within experimental uncertainty in some cases (the predicted values are within the experimental uncertainty of ± 0.05 in Chen et al. [87]), the sigma values are better than 0.05. Agreement within ± 0.05 logarithmic unit is classified as very good agreement, even in the systems that can be studies experimentally without difficulty [89]. As demonstrated by Wang et al. [2], dissociation quotients of water even at 25°C obtained by various researchers can differ by more than ± 0.05 in logarithmic unit. The above comparison demonstrates that predicted dissociation quotients of water in NaCl and KCl solutions are in satisfactory agreement with those determined by experimental studies. In addition, by using ε(H^+^, Cl^-^) and ε(K^+^, OH^-^) derived in this study, the dissociation quotients of heavy water (D_2_O) in 0.204 m KCl solution are also predicted from 50°C to 200°C, and compared with experimental data of Mesmer and Herting [86]. The predicted values are also satisfactory as indicated by a sigma value of 0.027.

**Table 10:** Comparison of dissociation quotient of water (log Q_w_) predicted by using SIT with experimental values

Experimental Data Sets*	Sigma Value σ
Harned and Owen [84]^A^: in 0.11–3.0 m_LiCl _at T = 25°C	0.023
Harned and Owen [84]^A^: in 0.11–3.0 m_KCl _at T = 25°C	0.034
Harned and Owen [84]^A^: in 0.11–3.0 m_NaCl _at T = 25°C	0.009
Mesmer et al. [85]^B^: in 1.0 m_KCl _at T = 25°C–200°C	0.038
Busey and Mesmer [83]^B^: in 0.1–5.0 m_NaCl _at T = 25°C–200°C	0.049
Mesmer and Herting [86]^B ^(heavy water): in 0.204 m_KCl _at T = 50°C–200°C	0.027
Chen et al. [87]^C^: in 0.1–5.0 m_NaCl _at T = 25°C	0.04

* Experimental data sets are listed in chronological order.

^A ^Experimental data set precise to three decimal places, cited in Baes and Mesmer [88].

^B^

Experimental data set precise to three decimal places.

^C ^Experimental data set precise to two decimal places.

Similarly, the experimental dissociation quotients of acetic acid in NaCl solutions at 25°C are compared with values predicted by using ε(H^+^, Cl^-^) and ε(Na^+^, Ac^-^) estimated in this study. In the comparison, log K° at 25°C is from Partanen and Covington [39]. The results in Table 11 indicate that predicted values agree with experimental values better than 0.020 in terms of sigma value.

**Table 11:** Comparison of dissociation quotient of acetic acid (log Q) in NaCl solutions predicted by using SIT with experimental values

Experimental Data Sets*	Sigma Value σ
Belevantsev et al. [90]^A^: in 1.0–4.0 M_NaCl _at T = 25°C	0.02
Robertis et al. [91]^A^: in 0.04–1.0 M_NaCl _at T = 25°C	0.01
Chen et al. [87]^B^: in 0.1–5.0 m_NaCl _at T = 25°C	0.019

*Experimental data sets are listed in chronological order.

^A^

Experimental data set precise to two decimal places.

^B ^Experimental data set precise to three decimal places.

To test the interaction coefficient of ε(GdAc^2+^, Cl^-^), ε(GdAc^2+^, Cl^-^) is used as an analog to ε(LaAc^2+^, Cl^-^), ε(NdAc^2+^, Cl^-^) and ε(YbAc^2+^, Cl^-^) to predict the first formation quotients of La^3+^, Nd^3+^, and Yb^3+ ^with Ac^- ^up to 70°C (log Q_1_), and then compare them with the respective experimental values of Ding and Wood [36]. It should be noted that those experimental results have not been employed to estimate any interaction coefficients. In calculations, the respective log β°_1 _at infinite dilution are from Ding and Wood [36], and ε(Gd^3+^, Cl^-^) is utilized as an analog to ε(La^3+^, Cl^-^), ε(Nd^3+^, Cl^-^), and ε(Yb^3+^, Cl^-^). The comparison indicates that predicted values are in very good agreement with experimental values. For log Q_1 _of LaAc^2+^, the maximum experimental uncertainty is 0.32 in comparison with the sigma value of 0.11 (Table 12). For log Q_1 _of NdAc^2+^, the maximum experimental uncertainty is 0.27 in comparison with the sigma value of 0.07 (Table 12). For log Q_1 _of YbAc^2+^, the maximum experimental uncertainty is 0.14 in comparison with the sigma value of 0.11 (Table 12)

**Table 12:** Comparison of formation quotients of REE with acetate (log Q_1_) in NaCl solutions predicted by using SIT with experimental values

Experimental Data Sets	Sigma Value σ
Ding and Wood [36]: formation quotients of LaAc^2+ ^in 0.1–2.0 m_NaCl _at T = 25°C–70°C	0.11
Ding and Wood [36]: formation quotients of NdAc^2+ ^in 0.1–2.0 m_NaCl _at T = 25°C–70°C	0.07
Ding and Wood [36]: formation quotients of YbAc^2+ ^in 0.1–2.0 m_NaCl _at T = 25°C–70°C	0.11

### Applications

It is well known that the determination of pH or hydrogen ion concentrations (pcH) is important and problematic at 100°C and higher temperatures because of the inapplicability of glass electrodes under these conditions. The above examples of model verification may point to the potential of using the solubility of brucite as a pH (pcH) buffer/sensor in experimental studies in NaCl solutions up to 5.0 m or up to the saturation of halite at temperatures up to 200°C. The advantages in using the brucite solubility as a pH (pcH) buffer/sensor include (1) its relatively fast kinetics to reach solubility equilibrium even at the room temperature [e.g., [79,80]], and (2) concentrations of magnesium are present at such a level that they can be analyzed with high precision by using the modern analytical instruments such as ICP-AES.

The governing equations for the solubility of brucite as the pH (pcH) buffer/sensor are Reactions (23) and (28) presented above in combination with the following charge and mass balance equations:





The first hydrolysis quotients of magnesium(II) at elevated temperatures in NaCl solutions can be taken from Palmer and Wesolowski [92]:

Mg^2+ ^+ H_2_O = MgOH^+ ^+ H^+ ^        (34)

To facilitate such applications, solubility quotients of brucite in 0.1–5.0 m NaCl solutions represented by Eq. (25) are predicted from 100°C to 200°C at P_SAT _at the increment of 50°C according to the interaction coefficients evaluated by this study (Table 13). When using brucite solubility as a pH buffer/sensor, it is advisable to utilize brucite of high-purity, because there are impurities in some commercially available brucite, which can result in non-stoichiometric dissolution, as noticed by Altmaier et al. [79] at 25°C.

**Table 13:** Predicted solubility quotient of Mg(OH)_2 _(s) (log Q_2_) in NaCl solutions at 100°C, 150°C and 200°C at saturated vapor pressures

Temperature, C	m_NaCl_	log Q_2_
100	0.1	13.44
	0.5	13.60
	1.0	13.68
	1.5	13.77
	2.0	13.82
	2.5	13.86
	3.0	13.90
	3.5	13.94
	4.0	13.98
	4.5	14.02
	5.0	14.05

150	0.1	11.63
	0.5	11.80
	1.0	11.88
	1.5	11.97
	2.0	12.02
	2.5	12.06
	3.0	12.09
	3.5	12.12
	4.0	12.16
	4.5	12.19
	5.0	12.22

200	0.1	10.22
	0.5	10.42
	1.0	10.51
	1.5	10.59
	2.0	10.64
	2.5	10.67
	3.0	10.70
	3.5	10.73
	4.0	10.75
	4.5	10.77
	5.0	10.80

## Conclusion

The SIT model, because of its less parameterized nature, is inherently less precise in reproduction of highly precise activity (and osmotic) coefficient data than the more parameterized Pitzer model, especially in high ionic strength region, even though it is advantageous in its mathematical simplicity. Therefore, the Pitzer formalism is the preferred method in treatment of activity (and osmotic) coefficients and other thermodynamic properties with high precision. However, in treatment of equilibrium constants, which are less precise than activity (and osmotic) coefficients in nature, the SIT model has the reasonable accuracy comparable to the Pitzer formalism. Hence, the SIT model would be a reliable method in evaluation of medium effects on thermodynamics, including its usage in extrapolation of equilibrium constants to infinite dilution, at elevated temperatures. It would be especially useful to experimental aqueous geochemists and chemists to assess the medium effect beyond ionic strength of ~1.0 m, which is the limit valid to the currently often employed B dot equation.
